# Letters on Syphilis

**Published:** 1854-01

**Authors:** 


ART. VIII. — *Letters on Syphilis.* Addressed to the chief editor of L'Union Medicale. By P. H. RICORD, Chirurgien de l'Hopital du Midi, etc., etc. Translated by D. D. SLADE, M. M. S., etc., etc. Boston: Printed by David Clapp.

This volume is a collection of those translations which appeared, from time to time, during the past year, in the pages of the Boston Medical and Surgical Journal.

It is said that the style of M. Ricord is one very original, abounding in idiomata, and of course very difficult to translate. This is a comforting assurance to that large class who have no especial fondness for the peculiarities of our author. We think, however, that the translator has sufficiently succeeded in rendering into English the somewhat luxuriant verbiage of his original. We see no necessity for apologies on that score. Youthful students of medicine will read it with avidity for the sake of the very spicy anecdotes with which it abounds.

Aside from its ornate style—its ornaments drawn from singular sources —this work is one of real and intrinsic value. It analyzes the causation and prophylaxis of syphilis, together with its modes of propagation, in a very able manner. Dr. Slade has added an analysis of the subject matter of the letters, which is brief, comprehensive, and intelligible. There is also an appendix which contains the formulæ of the Venereal Hospital at Paris.

For sale by DERBY, ORTON & MULLIGAN. S. B. H.

